# nCas9-based method for rolling-circle DNA substrate generation

**DOI:** 10.1016/j.ab.2025.115883

**Published:** 2025-04-25

**Authors:** Nischal Sharma, Kelsey S. Whinn, Harshad Ghodke, Antoine M. van Oijen, Jacob S. Lewis, Lisanne M. Spenkelink

**Affiliations:** Molecular Horizons and School of Chemistry and Molecular Bioscience, University of Wollongong, Wollongong, NSW, 2522, Australia

**Keywords:** Nickase Cas9, Guide RNAs, Rolling-circle DNA, Replication, Single-molecule imaging, Nucleic-acid biochemistry

## Abstract

Rolling-circle DNA replication is a DNA-duplication mechanism whereby circular DNA templates are continuously copied to produce long DNA products. It is widely used in molecular diagnostics, DNA sequencing, nanotechnology, and *in vitro* DNA replication studies. The efficiency of rolling-circle replication reaction heavily relies on the quality of the rolling-circle DNA template. Existing methods to create rolling-circle DNA substrates often rely on unique restriction sites and have limited control over replication fork topology and position. To address these limitations, we present a straightforward, customizable, and efficient strategy for producing rolling-circle DNA substrates with control over gap size and fork position. Our method relies on the use of nickase Cas9 (nCas9), which can be programmed to target specific DNA sequences using guide RNAs. In a one-pot reaction, we target nCas9 to four sites on an 18-kb plasmid to create 8–11-bp fragments. These fragments are removed and a flap oligo is ligated, to construct a fork with precisely controlled flap length and gap size. We demonstrate the application of this DNA substrate in an *in vitro* single-molecule rolling-circle DNA-replication assay. With our method, any plasmid DNA can be converted into a rolling-circle template, permitting generation of more physiologically-relevant DNA templates.

## 1. Introduction

**Fig. 1.**
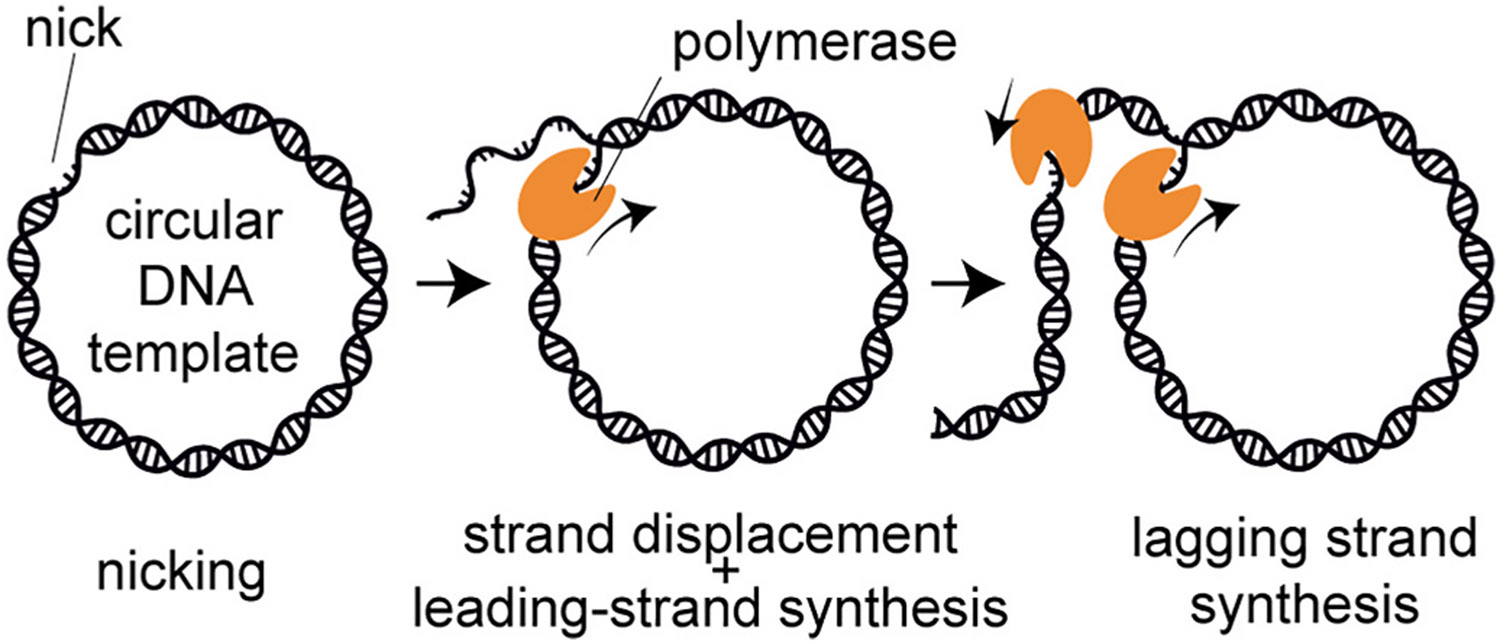
Schematic representation of rolling-circle DNA re**plication.** Plasmid DNA is nicked, allowing for the DNA polymerase to bind. The leading strand is produced through strand-displacement synthesis. The newly-synthesized strand acts as the template for lagging-strand synthesis.

Rolling-circle replication is a mechanism of DNA replication first described in bacteriophage φX174 [1,2]. Through rolling-circle replication, circular DNA templates are continuously copied to produce long DNA products (Fig. 1). In bacteriophages, this process begins with the nicking of one strand of the circular double-strand plasmid [3]. Next, a DNA polymerase binds to the nick and uses the 3′-hydroxyl group as a primer to initiate synthesis. The polymerase uses the intact strand as a template, while displacing the nicked strand as it proceeds around the circle [3,4]. As replication progresses, the continuously synthesized single-stranded DNA strand, the leading strand, is displaced and extruded. This newly-synthesized DNA serves as a template for lagging-strand synthesis in the form of short segments known as Okazaki fragments [4].

Rolling-circle DNA replication is generally processive and isothermal. Therefore, it has been used for molecular diagnostics, DNA sequencing, and nanotechnology [5-7]. Furthermore, rolling-circle replication has played a pivotal role in studying the fundamental mechanisms that underlie DNA replication [3]. The application of rolling-circle replication in single-molecule studies has enabled real-time visualization of individual replicating DNA molecules [8-10] and provided detailed insights on the diverse behaviors of individual replication components from both viruses [11] and prokaryotes [12,13].

The efficiency of rolling-circle replication in these applications requires highly purified undamaged DNA. These templates typically consist of a gapped double-stranded plasmid with a 5′-unpaired single-stranded overhang. Rolling-circle DNA substrates have been created through three main methods. One method starts by annealing a partially-complementary oligonucleotide fork to one end of the single-stranded plasmid [14]. The single-stranded plasmid is then converted to a double-stranded plasmid through primer extension by a DNA polymerase. This method relies on the efficiency of the DNA polymerase and has limited control over the exact gap size. Alternatively, rolling-circle substrates have been created through strand-displacement DNA synthesis at sites of nicks on double-stranded plasmid templates [15]. This method results in substrates lacking a gap at the fork and produce 5′-tails of variable lengths. The final method involves the creation of a single-stranded gap in a double-stranded plasmid, through the use of site-specific nicking enzymes [16]. While this method allows control over the gap size and flap length, it has limited flexibility to modify the fork position. Changing the fork position and gap size requires additional cloning steps to introduce or remove nickase recognition sites.

To address these limitations, we developed a method to create rolling-circle DNA substrates using the nCas9 enzyme, which allows for flexible manipulation of fork position and gap size. nCas9 is a modified version of the Clustered-Regularly-Interspaced-Short-Palindromic-Repeats (CRISPR)-associated protein 9 (Cas9) enzyme that can be programmed to target specific DNA sequences using guide RNAs (gRNAs) [17]. Due to the inactivation of one of its endonuclease domains, nCas9 nicks the DNA, unlike wild-type Cas9, which cuts the DNA. Here, we describe how we produce and validate a rolling-circle DNA template using 18-kb plasmid DNA. We show that this template enables efficient single-molecule rolling-circle replication.

## 2. Materials and methods

### 2.1. Reagents

#### Chemicals:

Acetone (Chem-Supply), agarose (Bioline), anti-digoxigenin-AP fab fragments (Life technologies), (3-Aminopropyl) triethoxysilane (ThermoFisher Scientific), ATP (Jena Bioscience), deoxynucleotidetriphosphates (dATP, dCTP, dGTP, dTTP) (Jena Bioscience), biotin-polyethyleneglycol (PEG) and mMPEG (MW 5000) (Laysan Bio), Bovine Serum Albumin (BSA, Sigma-Aldrich), dithiothreitol (DTT) (Astral Scientific), Ethylenediaminetetraacetic acid (EDTA, Sigma-Aldrich), ethanol (Chem-Supply), hydrogen chloride (Sigma-Aldrich), nucleoside triphospates (UTP, CTP, GTP, TTP) (Jena Bioscience), potassium glutamate (Sigma-Aldrich), magnesium chloride (Sigma-Aldrich), magnesium acetate (Sigma-Aldrich), neutravidin (ThermoFisher Scientific), sodium dodecyl sulfate (SDS, Sigma-Aldrich), sodium chloride (Sigma-Aldrich), sodium bicarbonate (Sigma-Aldrich), SYTOX Orange Nucleic Acid Stain (ThermoFisher Scientific), Tris (Astral Scientific), Tween-20 (Sigma-Aldrich).

#### Enzymes:

Nickase Cas9 (M0650S) (New England Biolabs), T4 DNA ligase (M0202S) (New England Biolabs), *Nae*I (R0190S) (New England Biolabs), *Eag*I-HF (NEB #R3505) (New England Biolabs).

***E. coli* DNA replication proteins** were purified previously: DnaB6 (DnaC)_6_ helicase-loader complex [18,19], the Pol III αεθ polymerase core [20], Pol III τ_3_δδ’ψχ clamp loader complex [20], β_2_ sliding clamp [21], single-stranded DNA-binding protein (SSB) [22] and DnaG primase [23].

#### Buffers:

Blocking buffer (1 × : 50 mM Tris-HCl (pH 7.9), 50 mM KCl, 2 % (*v/v*) Tween-20), *E. coli* replication buffer (1 × : 25 mM Tris-HCl (pH 7.9), 10 mM magnesium acetate, 50 mM potassium glutamate, 0.1 mM EDTA, 0.0025 % (*v/v*) Tween-20, 0.5 mg/mL BSA), Quenching buffer (2 × DNA Gel Loading Dye, 200 mM EDTA, 2 % (w/v) SDS), NEB buffer r3.1 (100 mM NaCl, 50 mM Tris-HCl, 10 mM MgCl2, 100 μg/ml Recombinant Albumin, pH 7.9 at 25 °C), NEB rCutSmart buffer (50 mM Potassium Acetate, 20 mM Tris-acetate, 10 mM Magnesium Acetate, 100 μg/ml Recombinant Albumin, pH 7.9 at 25 °C), TE buffer (10 mM Tris.HCl pH 7.6, 1 mM EDTA, 300 mM NaCl), Tris acetate EDTA buffer (TAE; 40 mM Tris, 20 mM acetic acid, 1 mM EDTA, pH 8.3).

### 2.2. Oligonucleotides

All PAGE-purified oligonucleotides were purchased from Integrated DNA Technologies (IDT, USA). The oligonucleotide sequences used for constructing the rolling-circle DNA substrates using one-pot and two-pot reactions are as follows in Tables 1 and 2.

### 2.3. Hybridization of nCas9-gRNAs

The hybridization of CRISPR RNA (crRNAs) and *Trans*-activating CRISPR RNA (tracrRNA) oligos was carried out as per IDT instructions in the following steps.

- RNA was resuspended in nuclease-free duplex buffer (IDT) to a final concentration of 100 μM.
- Equal concentrations of tracrRNA and crRNA were mixed to achieve a final concentration of 15 μM gRNA in nuclease-free duplex buffer. This mixture was heated at 95 °C for 5 min and then allowed to cool to room temperature overnight.
- Subsequently, equal concentrations of nCas9 and each gRNA were incubated in separate tubes at room temperature for 15 min, forming the different nCas9–gRNA complexes.

**Fig. 2.**
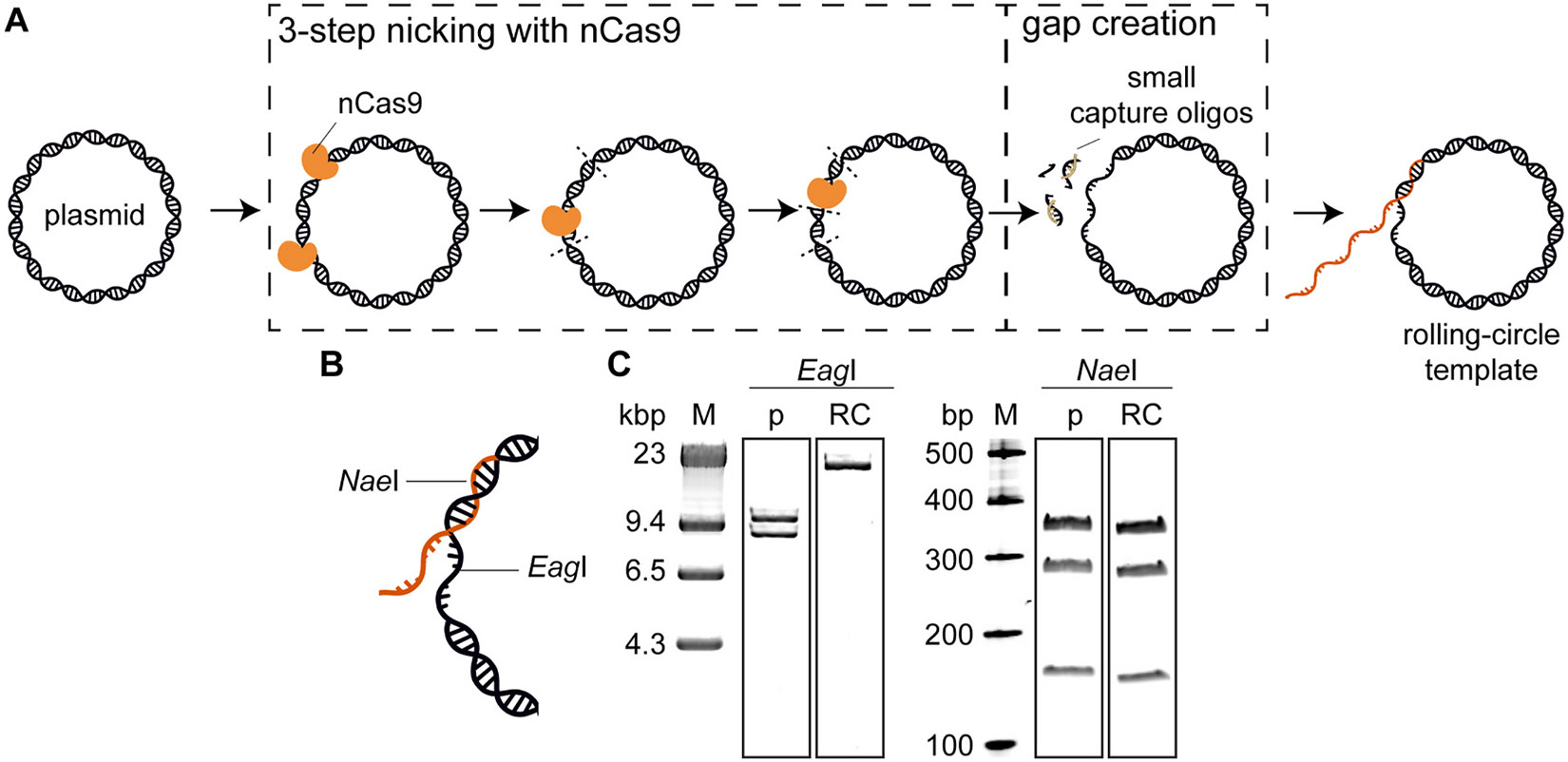
Construction and validation of the rolling-circle DNA **substrate.** (**A**) Schematic representation of the method. nCas9 is used to nick the DNA to create a gapped plasmid to which a fork oligo can be annealed and ligated. (**B**) Schematic representation showing the location of the *Nae*I and *Eag*I restriction sites used to validate the template. (**C**) Agarose gel showing the presence of the gap (left) and pre-cast gel showing the presence of the fork oligo (right). Full gel images are provided in Supplementary Fig. S2A-B.

While optimisation of crRNA–tracrRNA hybridization conditions is likely possible, the conditions used here gave 100 % nicking efficiency on our template (Fig. 2). Therefore, we did not explore this further.

### 2.4. Construction of the rolling-circle DNA template

We targeted nCas9 to create nicks in the plasmid DNA, creating 8–11-nt fragments. These fragments were removed by the addition of a large excess of capture oligos with complimentary sequence, resulting in a gapped plasmid. Finally, the fork oligo is annealed and ligated to create the rolling-circle template. All these steps were carried out in a one-pot reaction, without the need for purification in between steps.

The map of the 18-kbp pUBER plasmid, as previously described by Mueller et al., 2020 (27), and the specific nicking sites for gRNAs to introduce a DNA gap for the attachment of a biotinylated oligo are provided in Supplementary Fig. S1A-B.

- *Nicking*: The nicking reaction was carried out in three sequential steps. First, 50 μg (42 nM) of pUBER plasmid was treated with a 10-fold excess of nCas9–gRNA1 and nCas9–gRNA4 (420 nM each, Table 1) in 1 × NEB r3.1 buffer at 37 °C for 4 h in a thermal cycler, as per IDT instructions. The nicking reaction was then heat-inactivated at 75 °C for 10 min to denature nCas9 enzyme and gRNA molecules (omission of this step results in a reduction of overall efficiency). After allowing the reaction to cool down to room temperature for approximately 15–20 min, the second and third nicking reactions were carried out in the same way using nCas9-gRNA2 and nCas9-gRNA3 (Table 1), respectively. These subsequent reactions were carried out separately to prevent potential interference between nCas9-gRNA complexes.
- *Gapping*: A 50-fold molar excess of capture oligo-1 and capture oligo-2 was added to the nicking reaction in step i) and incubated at 80 °C for 20 min to displace the three small DNA fragments located between the two flanking nicked regions of plasmid DNA. Following this, the temperature was slowly decreased at a rate of 1 °C/min until it reached 16 °C to facilitate the annealing of the complementary capture oligos to the displaced oligos.
- *Annealing and ligation of the biotinylated Oligo*: A 100-fold excess of partially-complementary biotinylated oligonucleotide (with a 10-nt complementary sequence) was added to the reaction mixture in step ii) and incubated at 70 °C for 15 min. Annealing was achieved by slowly decreasing the temperature to 16 °C at a rate of 1 °C/min. The biotinylated oligonucleotide was ligated by adding 62.5 units/μg T4 DNA ligase, supplemented with 12 mM ATP and 10 mM DTT, once the temperature dropped to 37 °C. The reaction was then incubated at 16 °C for 18 h.
- *Purification*: Before purification, the ligase was inactivated by increasing the temperature to 65 °C for 10 min. The final forked DNA substrate was then purified from excess oligos (biotinylated fork oligos and hybridized capture and displaced oligos) using the gel-filtration chromatography as described previously [24]. Briefly, the annealing and ligation reaction from step iv) was loaded onto a Sepharose 4B column (1 × 25 cm; Sigma-Aldrich) equilibrated in gel filtration buffer (10 mM Tris-HCl pH 8.0, 1 mM EDTA, and 300 mM NaCl). The DNA substrate eluted as a single peak in the void volume. The final substrate was stored at −80 °C.

Target binding conditions can likely be optimised, for example by changing the magnesium concentration in the buffer. However, the conditions used here gave us 100 % nicking efficiency, so we did not explore target-binding optimizations. Any off-target nicks that may be introduced, will be re-ligated in subsequent steps and are therefore not of concern.

### 2.5. Verification

Correct assembly of the template was verified using the restriction enzymes *Eag*I and *Nae*I. The restriction digestion reaction was carried out by incubating 30–150 ng of the rolling-circle DNA substrate with 500 units/μg of either *Eag*I or *Nae*I in 1 × rCutSmart buffer at 37 °C for 2 h. The reactions were quenched by addition of stop solution (40 mM EDTA and 2 % (*w/v*) SDS) at a volume equal to the reaction volume.

DNA products from the *Eag*I digestion reactions, which generate larger DNA fragments, were separated using a 0.6 % (w/v) agarose gel in 1 × TBE buffer at 15 V for 800 min in a Wide Mini-Sub Cell GT System (Bio-Rad). The smaller DNA fragments generated by *Nae*I digestion were resolved on a 4–20 % Mini-PROTEAN TGX precast protein gel (Bio-Rad) in 1 × TBE buffer at 150 V for 75 min, using a Mini-PROTEAN Tetra vertical electrophoresis cell (Bio-Rad) (Refer to Supplementary Fig. S1A for the number of DNA fragments generated after *Eag*I and *Nae*I digestion reactions).The SYBR-Gold-stained gels were imaged on an Amersham Typhoon biomolecular imager (Cytiva).

If these verification steps indicate that the substrate was not formed correctly, optimisation of the gRNAs, the gapping, or the ligation might be required (see Supplementary Table 1 for more detail).

### 2.6. Single-molecule rolling-circle replication assay

To show that our DNA substrate can support DNA replication, we carried out *in vitro* single-molecule rolling-circle replication assays. Replication reactions were carried in microfluidic flow cells assembled using a Polydimethylsiloxane (PDMS) flow chamber placed on top of a glass coverslip functionalized with streptavidin-PEG-biotin, as previously described [12,14,18,24-27]. After assembly, we introduced with 300 μL of blocking buffer and incubated for at least 10 min to prevent nonspecific interactions of proteins and DNA with the flow-cell surfaces. The flow cell was then washed with 300 μL of 1 × replication buffer to remove the blocking buffer.

Single-molecule rolling-circle replication assays were carried out as describes previously [12], Briefly, 30 pM of rolling-circle DNA substrates was incubated with 15 nM DnaB_6_ (DnaC)_6_ in degassed 1X replication buffer in the presence of 1 mM ATP and 10 mM DTT at 37 °C for 2 min. Then, the DNA intercalating dye, SYTOX Orange (150 nM), was added and the reaction mixture was loaded into the flow cell at a constant rate of 10 μL/min to tether the DNA to the glass microscope coverslip via biotin–streptavidin–biotin linkage. Once an appropriate surface density of DNA was achieved, replication was initiated by loading of 30 nM αϵθ, 10 nM τ3δδ′ψχ, 46 nM β2, 75 nM DnaG, and 20 nM SSB, in 1X replication buffer, with 1 mM ATP, 10 mM DTT, 250 μM of each NTP, 50 μM of each dNTP, and 150 nM SYTOX Orange, at a constant flow of 20 μl/min.

### 2.7. Single-molecule imaging conditions

Single-molecule experiments were carried out using an Eclipse Ti-E inverted fluorescence microscope (Nikon, Japan) equipped with a CFI Apo TIRF 100× oil-immersion objective (NA 1.49, Nikon, Japan), as described previously [12,28,29]. The temperature of the flow cells was maintained at 31.2 °C using an electrically heated chamber (Okolab, USA). Images were captured using a 512 × 512 pixel^2^ EM-CCD camera (Andor iXon Life, EMCCD). NIS-Elements software (Nikon, Japan) was used to control the microscope, and the focus was maintained using the Perfect Focus System (Nikon, Japan). The DNA molecules were stained with 150 nM SYTOX Orange and imaged using a 532-nm laser (Coherent, Sapphire 532–200 CW) at 0.1 mW/cm^2^. The typical acquisition parameters were set at 1 frame/s for 4 min with an exposure time of 200 ms.

### 2.8. Analysis of single-molecule replication kinetics

Image analysis was carried out in ImageJ, v1.51w. First, raw acquisitions in nd2 format were corrected for beam profile and x,y drift. Next, replicating molecules of interest were selected and a kymograph, a graphical representation that shows the progression of replication forks along the DNA template over time, was generated for each molecule.

#### Rate analysis:

Here, replication rate refers to the speed at which the DNA molecule grows (*e.g.* the speed at which replication progresses). Positions of the tip of the replicating molecule were determined as previously described [25]. These coordinates were used to detect individual segments of constant rate. Finally, the data were fit to a Gaussian distribution function using MATLAB 2016b.

#### DNA length analysis:

Here, the DNA length refers to the total length of the replicated DNA product, after 2 min of imaging. The length of the DNA product was determined by measuring the final length of the 1D line (in pixels). Finally, the data were fit to a single exponential function using MATLAB 2016b.

## 3. Results and discussion

### 3.1. Template construction

We developed a method to generate rolling-circle templates using nCas9. First, the plasmid DNA is nicked in three steps to introduce four nicks (Fig. 2A). We add nCas9–gRNA complexes to create nicks at four locations (Fig. 2A, Supplementary Fig. S1B). The nicks at these four locations create three small DNA fragments. These small fragments are then displaced from the plasmid by increasing the temperature, before they are captured by adding complementary capture oligos (Fig. 2A). This process creates a 35-nt-long DNA gap on the plasmid, where the partially-complementary biotinylated fork oligonucleotide can be attached (Fig. 2A, Supplementary Fig. S1B). We achieve an overall efficiency of 47 ± 4 % (*N* = 3).

### 3.2. Validation

To verify correct assembly of the rolling-circle templates, we used two restriction enzymes to cut the template. *Eag*I is used to verify the presence of the gap (Fig. 2B). *Eag*I has two recognition sites within pUBER plasmid (Supplementary Fig. S1A). Therefore, restriction of pUBER with *Eag*I will result in two linear fragments (~8.5 kb and ~9 kb, respectively). For successfully-formed rolling-circle substrates, however, one recognition site falls within the gap and cannot be cleaved. Therefore, restriction of the correctly-formed rolling-circle substrate with *Eag*I is expected to produce an 18-kb linear fragment (Fig. 2B. Supplementary Fig. S1B). Fig. 2C (left) shows that treatment with *Eag*I produces one linear fragment, indicating all the templates contain a gap. *Nae*I was used to confirm successful ligation of the fork oligonucleotide to the DNA substrate (Fig. 2B). *Nae*I cleaves the plasmid DNA and the rolling-circle template at five sites (Supplementary Fig. S1A), resulting in five fragments (~12 kb, 6 kb, 354 bp, 283 bp 160 bp). If, however, the fork is not successfully annealed and ligated, one of these sites is located in the gap and will not be cut (Supplementary Fig. S1B). As a result, digestion of unsuccessfully-created substrates with *Nae*I would not show the 160-bp and 354-bp bands. Fig. 2C (right) shows the three lowest bands of the *Nae*I-restricted plasmid and rolling-circle template on a precast gel. All three bands are present, indicating successful annealing and ligation of the fork oligo.

### 3.3. Single-molecule visualization

**Fig. 3.**
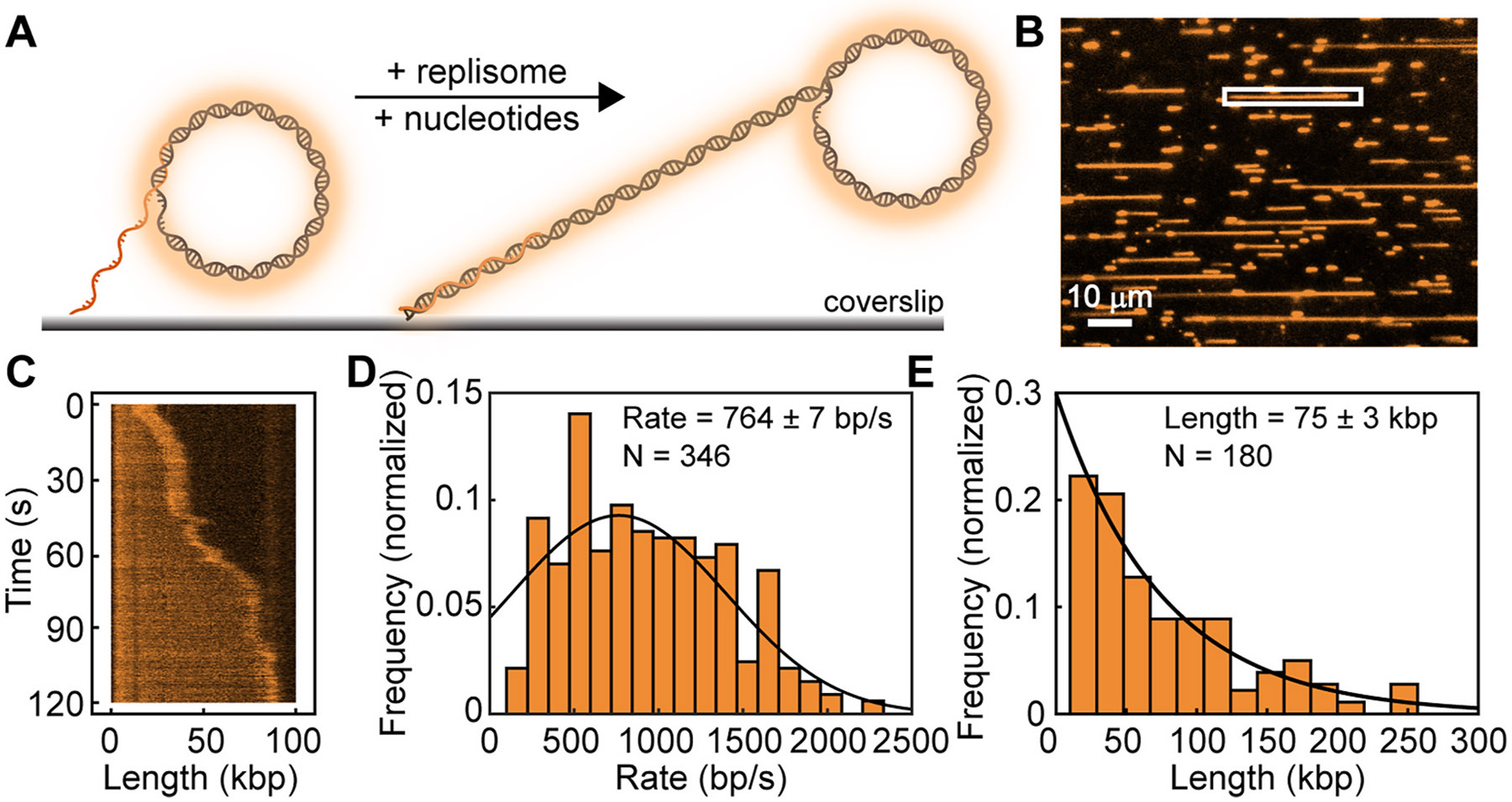
Single-molecule visualization of rolling-circle re**plication.** (**A**) Schematic representation of the single-molecule rolling-circle assay. Rolling-circle templates are immobilized on the surface of a microscope coverslip in a microfluidic flow cell. Replication is initiated by loading all the replisome components. Replication products are stretched out by laminar flow, and visualized using the intercalating dye SYTOX Orange. (**B**) Typical field of view showing rolling-circle replication products stretched out in the direction of flow (left–right). Scale bar is 10 μm. (**C**) kymograph of the boxed molecule in (B). Tracking of the position of the tip of the DNA allows quantification of replication rate and final product length. (**D**) Histogram of the instantaneous rate of replication. A Gaussian-distribution fit to the data (black line) gives a rate of 764 ± 7 bp/s (mean ± s.e.m., *N* = 346). (**E**) Histogram of the final product length. The black line represents a single-exponential fit giving a length of 75 ± 3 kbp (mean ± s.e.m., *N* = 180).

To demonstrate the use of this template, we chose a single-molecule rolling-circle assay using the *E. coli* DNA replication system. Comprehensive analysis of this reaction has been done previously [8,25,26,30-36]. We simply use this system to demonstrate that our substrate supports replication with similar efficiencies to other rolling-circle templates. We loaded our template onto the surface of a microscope coverslip in a microfluidic flow cell in the presence of SYTOX Orange. We then added all the purified *E. coli* replisome components to initiate replication. The laminar flow of buffer will stretch the newly-synthesized DNA in the direction of flow (Fig. 3A) allowing visualization though SYTOX Orange. Fig. 3B shows a typical field of view, where each line represents a replication product. Our rolling-circle template has a replication efficiency (percent of templates that are replicated) of 44 ± 4 %, similar to efficiencies reported for other substrates [19]. We can monitor replication of individual templates over time (Fig. 3C), allowing us to measure the instantaneous rate of replication [25] and the final product length after 2 min for all molecules. The rate (764 ± 7 bp/s) and product length (75 ± 3 kbp) are the same as previously reported rates for rolling-circle replication by the *E. coli* replisome [34].

## 4. Conclusion

In summary, we present a straightforward, customizable, and efficient strategy to generate rolling-circle DNA substrates with direct control over the gap size and fork position (Table 2). Our method has comparable cost and time requirements to existing approaches (Table 2). We show that these template support robust DNA replication, with similar efficiencies and rates to other previously reported rolling-circle templates.

Gap size is known to affect the efficiency of replisome assembly [45]. Our method can be used to produce optimal gap sizes for different replisomes. Control over the fork position can be used to provide spatial separation between the fork and a genetic element of interest, such as transcription promotors or site-specific DNA damage [12]. These templates, therefore, allow studies on the behavior of the replisome upon encountering obstacles like secondary DNA structures, DNA-bound proteins, and lesions. Furthermore, control over the fork position enables the deliberate avoidance or targeting of regions with high secondary structure. While it is known that secondary structures can affect nCas9 nicking efficiency, methods have been developed to overcome these issues [46]. Thus, we anticipate that our method will allow production of rolling-circle templates for the study of replication protein dynamics on increasingly complex and crowded DNA substrates [47].

## Supplementary Material

Supplementary Information

## Figures and Tables

**Table 1:** Oligonucleotide sequences used for constructing the rolling-circle DNA substrate using one-pot reaction.

Biotinylated-fork oligonucleotide	5′-/bio/TT TTT TTT TTT TTT TTT TTT TTT TTT TTT TTT TTT TTT TTT TTT TTT TTT TTT TTT TTT TCA TGC CGG CG
Complementary capture oligo-1	TGG CGG CCG ACG C
Complementary capture oligo-2	CT GGG CTA CGT CT
tracrRNA	Product: Alt-R^®^ CRISPR-Cas9 tracrRNA, No: 1072532
crRNA1	5′ GAGAAGCAGGCCATTATCGC**CGG** 3′
crRNA2	5′ GGCCATTATCGCCGGCATGG**CGG** 3′
crRNA3	5′ GGCATGGCGGCCGACGCGCT**GGG** 3′
crRNA4	5′ CGCGCTGGGCTACGTCTTGC**TGG** 3′

**Table 2:** Comparison of rolling-circle substrate construction methods. Cost represents approximate cost based on prices listed by New England Biolabs and Integrated DNA Technologies.

Method	Primer extension	Strand displacement	Synthetic minicircle	Restriction gapping	nCas9 method
**Starting material**	ssDNA plasmid	dsDNA plasmid	Synthetic oligos	dsDNA plasmid	dsDNA plasmid
**Fork topology control**	Limited	Limited	Yes	No	Yes
**Flap length control**	Yes	No	Yes	Yes	Yes
**Time**	~4 h	~6 h	~48 h	~24 h	~24 h
**Yield**	Moderate (~50 %)	High (>90 %)	Low (~15-30 %)	Moderate (~50 %)	Moderate (~50 %)
**~Cost (USD)**	$250	$200	$500	$400	$400
**References**	[8,37-39]	[15,40]	[41-43]	[44]	

## Data Availability

Data will be made available on request.

## Abbreviations

CRISPR: Clustered regularly interspaced short palindromic repeats
Cas9: CRISPR-associated protein 9
nCas9: Nickase Cas9
crRNA: CRISPR RNA
tracrRNA: *Trans*-activating CRISPR RNA
gRNA: Guide RNA
PEG: polyethyleneglycol
BSA: Bovine Albumin Serum
DTT: dithiothreitol
EDTA: Ethylenediaminetetraacetic acid
SDS: sodium dodecyl sulfate
