# Supplementary material for: nCas9-based method for rolling-circle DNA substrate generation
Source: Anal Biochem. Author manuscript; Available in PMC 2025 Aug 1. (PMC12145885; doi:10.1016/j.ab.2025.115883)
Supplement: Supplementary Information [file NIHMS2086320-supplement-Supplementary_Information.docx]

# Supplementary Material


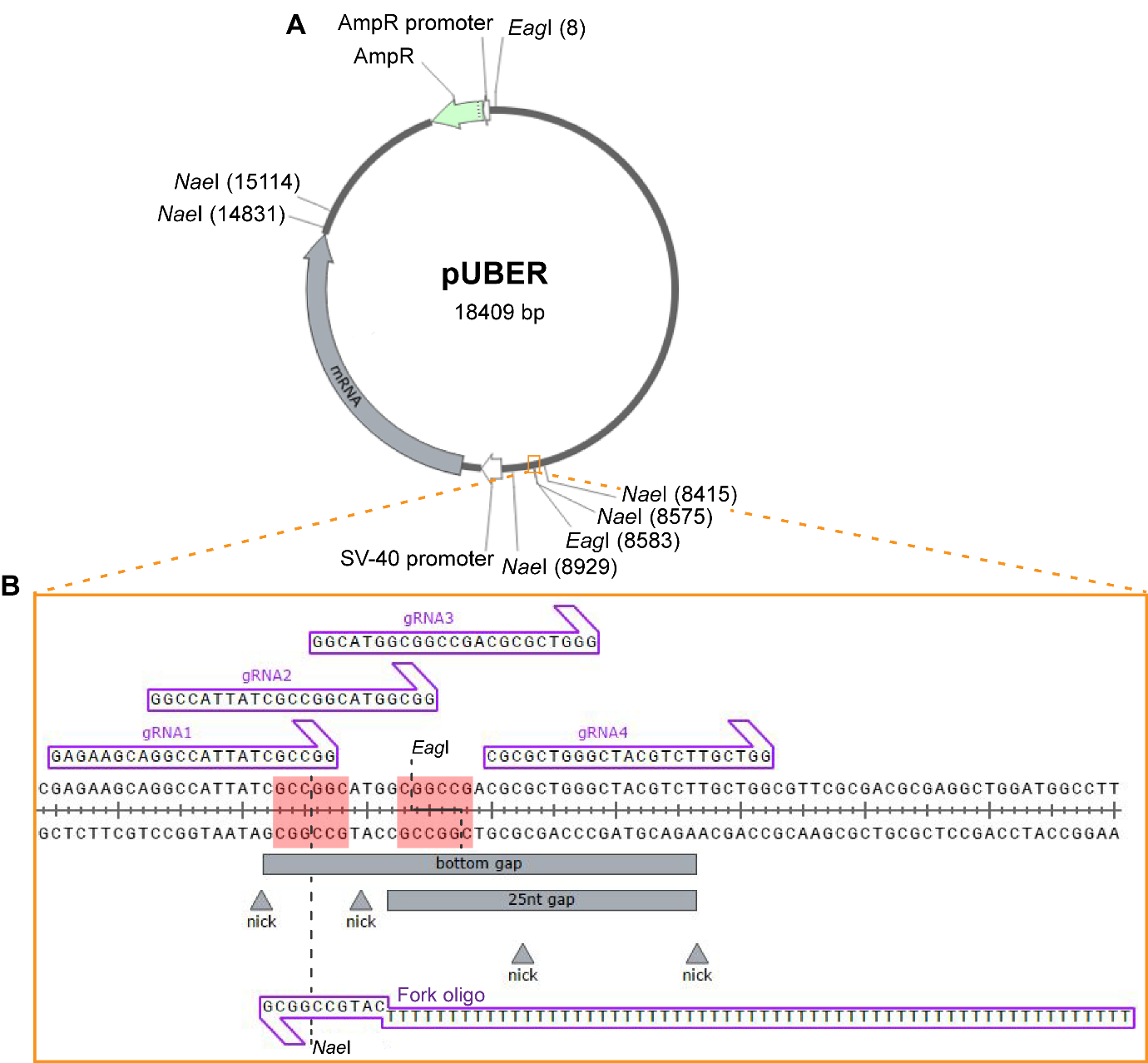


**Supplementary Fig. S1**. Simplified pUBER plasmid map with *Eag*I and *Nae*I restriction sites. (A) The circular pUBER plasmid map, showing two restriction sites for *Eag*I and five restriction sites for *Nae*I. *Eag*I digestion of the pUBER plasmid occurs at positions 8 bp and 8583 bp, resulting in two distinct DNA bands of 9834 bp and 8575 bp. *Nae*I digestion occurs at positions 8415 bp, 8575 bp, 8929 bp, 14831 bp, and 15114 bp, generating DNA fragments of 11710 bp, 5902 bp, 354 bp, 283 bp, and 160 bp, respectively. (B) A zoomed-in view of the DNA sequence at the gap site, showing the positions of all gRNAs for creating nicks, annealing biotinylated forks at the gap, and the *Eag*I and *Nae*I restriction sites (highlighted in red). If the rolling-circle template is successfully formed, one of the *Eag*I recognition sites will be located within the gap, resulting in an 18-kb linear fragment upon digestion. Additionally, if the fork is successfully annealed and ligated to the DNA gaps, *Nae*I digestion will produce three smaller DNA bands of 350 bp, 283 bp, and 160 bp.


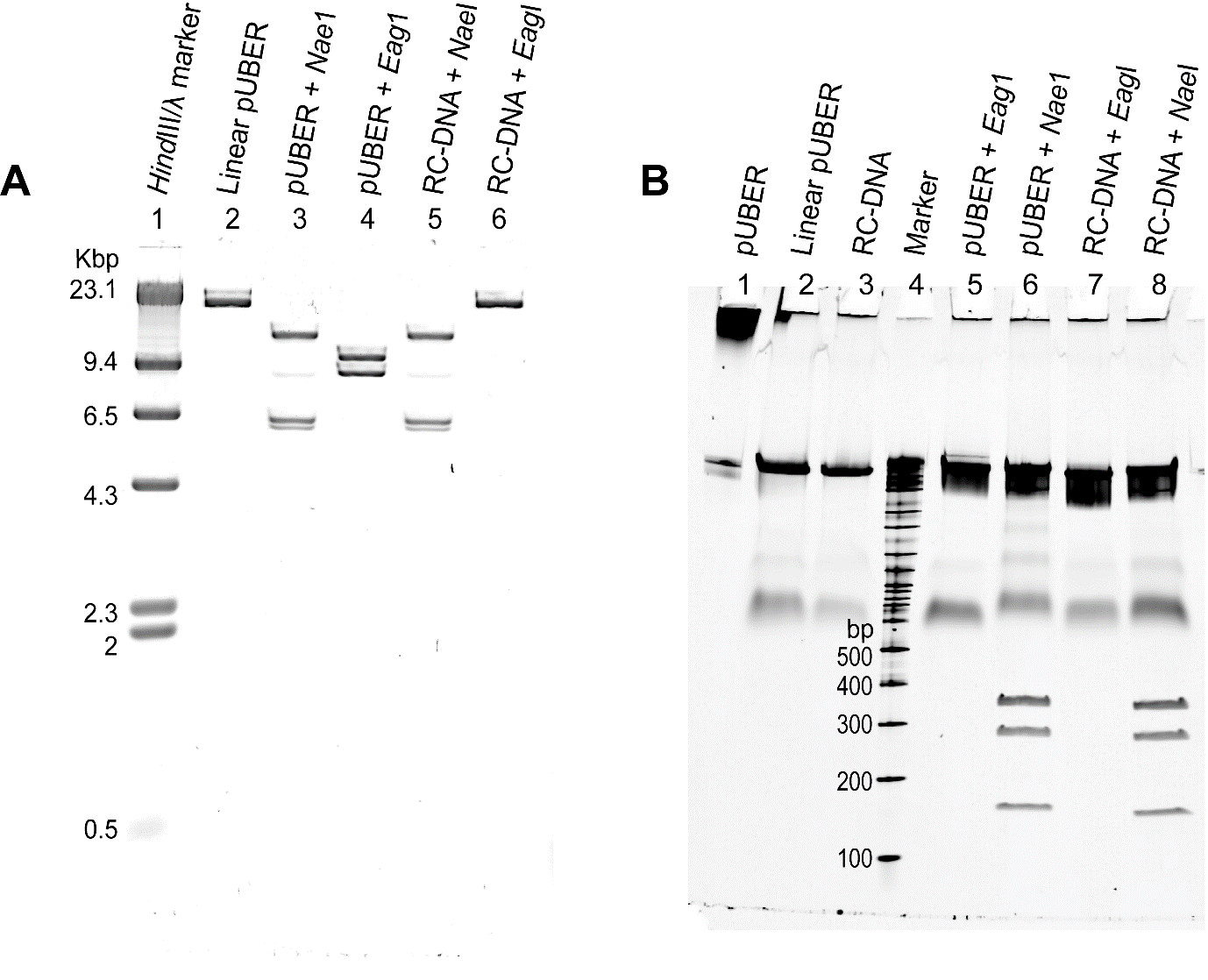


**Supplementary Fig. S2**. Uncropped gel analysis of *Eag*I and *Nae*I digestion reactions to verify DNA gaps and attachment of fork oligonucleotides within the rolling-circle DNA substrate. (A) Agarose gel (0.6% w/v) analysis of *Eag*I and *Nae*I digestion reactions for the circular pUBER control and forked rolling-circle DNA substrates. *Eag*I digestion of the circular pUBER DNA produced two distinct DNA bands (Lane 4). In contrast, *Eag*I digestion of the rolling-circle DNA substrate generated a single linear DNA band (Lane 6) similar to linear control (Lane 2), confirming the presence of a gap within the rolling-circle DNA substrate. *Nae*I digestion of the circular pUBER control and forked DNA substrate produced two DNA bands corresponding to the expected sizes of 11,710 bp and 5,902 bp (Lanes 3 and 5). The three additional smaller size DNA bands, 354 bp, 283 bp, and 160 bp, were detected using a 4-20% precast protein gel. (B) *Nae*I digestion of both the circular pUBER and rolling-circle DNA substrates produced three additional smaller DNA bands, sized 354 bp, 283 bp, and 160 bp (Lanes 6 and 8), indicating that the biotinylated fork oligos were efficiently ligated to the DNA substrate. *Eag*I digestion of pUBER (Lane 5) and the rolling-circle DNA substrate (Lane 7), as well as pUBER (Lane 1), linear pUBER (Lane 2), and rolling-circle pUBER (Lane 3), did not produce these smaller DNA bands.

**Supplementary Table S1:** Troubleshooting

| Incomplete nicking | Design gRNAs with High Specificity: Use computational tools for sgRNA optimization [[1](#_ENREF_1)], or optimize sgRNA length [[2](#_ENREF_2)]. |
| --- | --- |
| Incomplete fragment excision | Consider increasing the excess of capture oligos. Remove the captured fragments through a purification step to prevent reannealing. |
| Failed flap ligation | Potential lack of ATP in the ligation buffer. Consider supplementing with extra ATP.  Potential mismatched annealing. Consider annealing + ligation at a higher temperature, using a ligase that operates at higher temperatures (eg. Hi-T4 DNA ligase, NEB).  Include PEG8000 in the ligation buffer as it enhances hybridization and ligation rates [[3](#_ENREF_3)]. |

# References

1. Chen, Q., et al., *Genome-wide CRISPR off-target prediction and optimization using RNA-DNA interaction fingerprints.* Nat Commun, 2023. **14**(1): p. 7521.

2. Matson, A.W., et al., *Optimizing sgRNA length to improve target specificity and efficiency for the GGTA1 gene using the CRISPR/Cas9 gene editing system.* PLoS One, 2019. **14**(12): p. e0226107.

3. Yeung, P.Y., et al., *Systematic evaluation and optimization of the experimental steps in RNA G-quadruplex structure sequencing.* Sci Rep, 2019. **9**(1): p. 8091.
